# Epidemiological study of dysphonia in 4-12 year-old children

**DOI:** 10.1590/S1808-86942011000600010

**Published:** 2015-10-19

**Authors:** Elaine Lara Mendes Tavares, Alcione Brasolotto, Marcela Ferreira Santana, Carlos Alberto Padovan, Regina Helena Garcia Martins

**Affiliations:** 1 Speech and Hearing Therapist, PhD in Clinical Epidemiology, Speech Therapist – Otorhinolaryngology Program – Medical School of Botucatu; 2 PhD; Professor – Department of Speech and Hearing Therapy – University of São Paulo - Bauru. Professor – School of Speech and Hearing Therapy; 3 Medical Student – Medical School of Botucatu, UNESP; 4 Full Professor – Department of Biostatistics of the Biosciences Institute of UNESP. Professor – Department of Biostatistics; 5 Senior Associate Professor of the Medical School of Botucatu - UNESP. Had of the Phoniatrics and Voice Ward. Professor of Otorhinolaryngology of the Paulista State University - Unesp, Campus of Botucatu. Medical School of Botucatu (UNESP)

**Keywords:** child, epidemiology, voice disorders

## Abstract

**Abstract:**

Children dysphonia studies have reported an incidence of 4.4 to 30.3%.

**Goals:**

To establish the prevalence of dysphonia in children, based on the opinion of the parents, acoustic and vocal-perceptual assessments, associated symptoms, risk factors and videolaryngoscopy findings.

**Materials and Methods:**

The parents from 2,000 children answered a questionnaire about the vocal quality of their children, and these children were submitted to perceptual vocal, acoustic and videolaryngoscopy assessments.

**Results:**

We had 1,007 boys and 993 girls; sporadic symptoms were reported by 206 parents and permanent symptoms were reported by 123. In the perceptual assessment, the G parameter (degree of dysphonia) had a score of 0 in 694 voices; 1 in 1,065 and 2 in 228. There was f0 reduction with age and the remaining acoustic parameters were high in children with a G score of 2. Nodules, thickening and inflammation were the most common in the videolaryngoscopy exams.

**Conclusions:**

Parental judgment indicated a prevalence of dysphonia in 6.15%, and perceptual analysis yielded a value of 11.4%. Vocal symptoms were associated with a phonatory overload. sinonasal disorders, vocal abuse and noise were considered relevant risk factors. The acoustic analysis kept a direct association with the perceptual-auditory. Laryngeal lesions were found in the videolaryngoscopy exams, stressing nodules, thickening and inflammation.

## INTRODUCTION

Epidemiological studies on pediatric dysphonia are rare in the literature and the difficulties in doing them have been reported by numerous authors, such as the very definition of the word “dysphonia”^1^,^2^. Moreover, vocal utterance from children bear aspects which should not be considered pathological, stressing the mild instability and breathiness – due to the neuromuscular immaturity of laryngeal structures and the posterior triangular slit, respectively - a known characteristic of children glottic configuration. Effort, stress and pitch elevation during vocal utterance are also frequently seen in children, especially during recreational activities. These vocal characteristics are frequently interpreted as abnormal and overrated by the parents; on the other hand, in other situations, parents do not perceive these symptoms as representing vocal disorders in their children, which impairs the answers in the assessment questionnaire; thus, delaying diagnosis. To these factors we add the parents' resistance in taking their children to endoscopic exams, deemed decisive for diagnostic purposes. The technical difficulties in exposing the larynx during videolaryngoscopic exams and the lack of collaboration from the children in these procedures are factors which impair even further the feasibility and quality of the studies^1^, ^2^, ^3^.

The aforementioned reasons justify the highly variable rates of children dysphonia prevalence reported in the literature - between 4% and 30%^4^, ^5^, ^6^, ^7^, ^8^, ^9^, ^10^, ^11^. The lower rates pointed out by some authors are, often times, calculated solely based on the assessment questionnaires filled out by the parents, which are not always reliable; on the other hand, higher rates may represent physiological vocal disorders, which are inherent to this age range. Moreover, some epidemiological studies on this topic are not very rigorous in patient recruitment and in the make up of the groups, and they do not investigate the comorbidities directly associated to the development of dysphonia^12^,^13^. Then, it is clear the importance of a detailed critical analysis of epidemiological studies for the proper interpretation of the results, as well as the use of other assessment tools, not being restricted only to the content of the questionnaires. Having said that, many studies on dysphonia have utilized the GRBASI^3^,^14^,^15^ scale for the auditory-perceptual analysis, which is considered an excellent method for vocal assessment, especially when employed by experienced professionals. Computerized vocal acoustic analysis has been included in the studies in order to provide complementary quantitative registers to the assessments and the video-endoscopies help clear up laryngeal diagnoses.

The goals of the present study were: to establish the prevalence of dysphonia in children between 4 and 12 years of age from the city of Botucatu public school system, in the state of São Paulo, based on parental judgment, auditory and acoustic perceptual assessments, analysis of associated vocal symptoms, risk factors and videolaryngoscopic findings.

## MATERIALS AND METHODS

**Figure 1:**
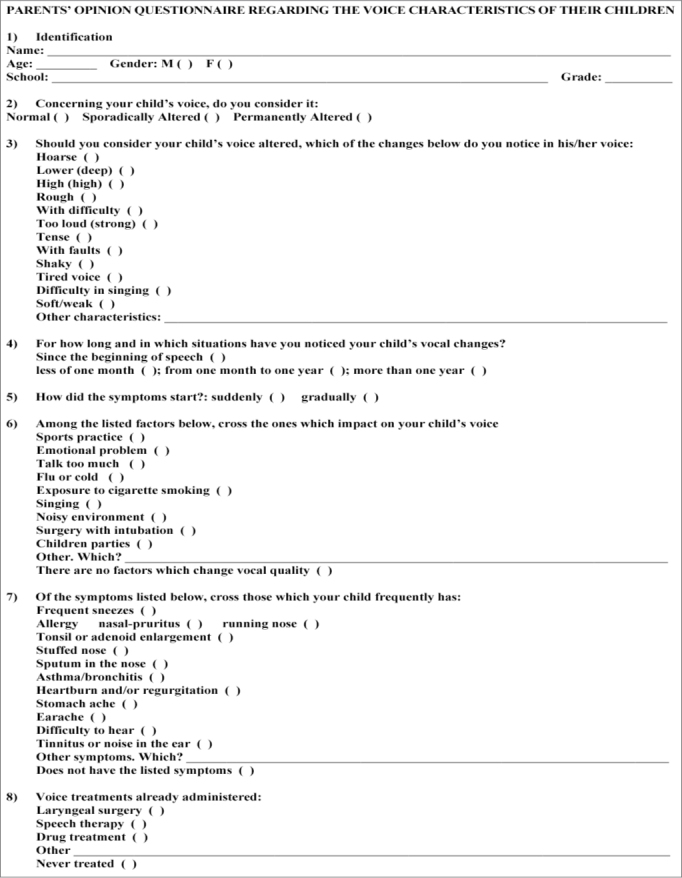
Pediatric dysphonia questionnaire distributed to the parents.

After being approved by the Ethics in Research with Human Beings Committee of a university institution (process # 2136/2006) and the City Secretary of Education, we distributed 2,700 questionnaires to the parents of the children enrolled in the eight public schools of the city were the study was carried out, chosen at random from the 29 schools in the city; and accompanying the questionnaire there was also an informed consent form – previously approved by the Ethics Committee of the Institution, under the same process # aforementioned. Most of the questions in the questionnaire were multiple-choice, in order to facilitate the answers of the parents and analysis of the results, and there was only a handful of open questions (Figure 1). The parents were also given a document with definitions of normal and altered voice and were invited to participate in some meetings to clear up their doubts and to receive general instructions on how to fill out the questionnaires.

When the parents returned the questionnaires, that showed interest and compliance towards the study. Those children with incomplete questionnaires (n-630) or those children in whom it was not possible to carry out the speech and hearing assessments (n-70) were taken off the study. Thus, we selected 2,000 children; who were then divided in three age ranges: between 4 and 6 years; 7 and 9 years; 10 and 12 years. The questionnaires had the following data: identification; the presence of vocal disorder; vocal symptoms; time and shape of vocal symptom onset and associated factors; and treatments for the vocal disorder. The exclusion criteria utilized were: belonging to an age range different from the one established; reporting a hearing disorder; genetic syndrome and/or craniofacial malformation; having a past of prolonged intubation or neck trauma; having neurological disorders with voice and speech involvement.

The 2,000 children were taken to speech and hearing acoustic and auditory-perceptual assessments, which were carried out in a silent room at the schools. All the children were also referred to videolaryngoscopy, which was carried out in the Otorhinolaryngology Ward of the Federal University of São Paulo in Botucatu - UNESP, Botucatu. In order to calculate the dysphonia prevalence indices, based on the reports from the parents, we considered only the permanent or frequent vocal symptoms, disregarding sporadic vocal complaints.

We used the GRBASI scale for the auditory-perceptual assessment, which is based on the following parameters: G (Grade of change), R (Roughness), B (Breathiness), A (A*sthenia*), S (Stress) and I (Instability). The recordings were made during spontaneous speech, counting numbers and sustained utterance of the (/a/) vowel; and the scale was employed by three experienced voice-experts; and there had to be an agreement among at least two of them. The voices were considered dysphonic when assigned scores higher than 1 in the G parameter from the GRBASI scale.

We used the MDVP (Multi-Dimensional Voice Program – Multi Speech 3700, model 5105, from Kay Elemetrics Corporation, Germany) system for the acoustic vocal analysis, coupled to a microcomputer, with a standard sound board (Soundblaster). The vocal samples were captured by a headset microphone (Shure-USA) connected to a sound mixer (Xenyx 502, from Behringer– Germany) during the sustained utterance of the /a/ vowel, maintaining confortable frequency and intensity; and for that, the children were previously trained. In order to carry out the analyses, we discarded the initial and final two seconds of the recordings, for they could bear utterance instability. The following parameters were considered: Fundamental Frequency (f_0_), Percentage of *Jitter* (%), *Pitch Perturbation Quotient* (*PPQ-*%), *Shimmer Percentage* (%), *Amplitude Perturbation Quotient* (*APQ* -%), *Noise Harmonic Ratio (NHR) and Soft Phonation Index (SPI)*.

Among the 2,000 children enrolled in the videolaryngoscopy exams, there were only 259 who came for testing; and of these, 222 were examined using the rigid telescope (70^o^, 8mm, from Asap, Germany), and 37 with the flexible nasofibroscope (3.3mm, Olympus, Japan), for they did not allow the telescopic exam. In order to capture images, we used the multifunctional video system type XE-50 - Eco V 50W X -TFT/USB – ILO ELETRONIC GnbH – Carl – Zeiss, Germany, tandem system.

Videolaryngoscopic diagnoses were defined as follows:

• **Vocal nodules –** bilateral sessile lesions, clear, symmetric, on the free border of the vocal folds, at the joint between the anterior third with the middle third of the glottic phonation area;
• **Vocal polyps –** sessile or pedicled, unilateral lesions (rarely bilateral), gelatinous and mobile when pedicled;
• **Mucosal or epidermal cysts –** the lesions were unilateral, circumscribed, well-outlined, round, with mucus content (mucous cyst) or caseous (epidermal cyst). When the cyst content was drained through an opening in the mucosa, it characterized the **fistulized cyst**;
• **Mucosal bridge –** mucosal arch along the vocal fold, with variable extension and width;
• **Sulcus vocalis –** dark linear lesion on the vocal fold, parallel to the free border, uni or bilateral, varying in depth and extension (**major or minor sulcus stria**). When the mucosal depressions were restricted to a small region, keeping the bottom epithelized, it was called a **pocket-type sulcus**;
• **Anterior laryngeal micro membrane –** micro diaphragm joining the anterior region of the vocal folds with the glottic or infraglottic insertion;

Besides the aforementioned lesions, we also considered other inflammatory changes of the laryngeal mucosa, such as: edema, hyperemia and mucosal epithelial thickening. Some endoscopic diagnoses are difficult to differentiate (such as: the pocket-type sulcus, fistulized cyst, mucosal bridge), were confirmed during direct laryngoscopy.

For the statistical analyses, in the characterization of the symptoms/behavior; type of voice, associated factors and the vocal symptoms of the children, we built a 95% confidence interval using the Goodman Homogeneity Test. The variance analysis was used for the following variables: vocal symptoms reported by the parents, gender, age range, G score and acoustic parameters. All discussions were held at the 5% significance level.

## RESULTS

**Figure 2:**
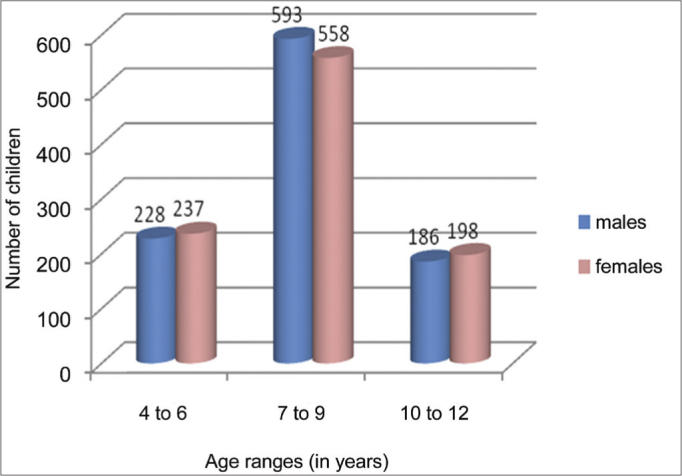
Distribution of the children in gender and age range.
• **Parental judgment as to their children's vocal symptoms, distributed in gender and age ranges (years)**: when asked if they considered the voices of their children as normal or changed, 1,671 of them (83.6%) did not report any vocal symptom; 329 parents reported yes – sporadic symptoms in 206 children (10.3%) and frequent or permanent in 123 (6.15%). The results from these assessments are depicted on Table 1.

• **Gender and age ranges (in years)**: we had 2,000 children in the study, distributed in the age ranges depicted on Figure 2.

**Table 1:** Parents' reports on the vocal symptoms according to gender and age range (in years).

Vocal symptoms		Absent		Sporadic		Permanent		Total
Gender	Age range	N	%	N	%	N	%	
	4-6	196	86.0 a(1)A(2) β(3)	17	7.4 aAα	15	6.6 aAα	228
Male	7 - 9	477	80.4 aAγ	83	14.0 aAα	33	5.6 aAβ	593
	10 - 12	153	82.3 aAβ	15	7.5 aAα	18	10.2 aAα	186
	4 - 6	202	85.2 aAβ	21	8.9 aAα	14	5.9 aAα	237
Female	7 - 9	478	85.7 aAγ	53	4.8 aAα	27	9.5 aAβ	558
	10 - 12	165	83.3 aAβ	17	8.1 aAα	16	8.6 aAα	198
Total		1.671	83.60	206	10.30	123	6.15	2.000

(1) Two percentage frequencies followed by the same low-case letter are not different as to respective age ranges (lines), when we fix the gender (*p*>0.05).

(2) Two percentage frequencies, followed by the same upper-case letter do not differ as to the respective genders (columns), when we fix the age range (*p*>0,05).

(3) Two percentage frequencies followed by the same Greek letter do not differ as to the respective symptoms, when we fix gender and age range (*p*>0,05).
• **Characterization of vocal symptoms/behaviors reported by the parents**: Table 2 lists the main vocal symptoms reported by the parents and the corresponding confidence thresholds, stressing hoarseness and fatigue upon speaking.

**Table 2:** Confidence threshold according to voice characterization as per perception from the parents.

		Confidence threshold (95%)	
Type of voice	Absolute frequency (proportion)	Lower limit	Upper limit
Adequate	1598 (0.799)	0.781	0.817
Hoarse	152 (0.076)	0.064	0.088
Tired voice	104 (0.052)	0.042	0.062
High/acute	101 (0.051)	0.041	0.060
Low/deep	72 (0.036)	0.028	0.044
Strong/intense	35 (0.018)	0.012	0.023
Difficulty to sing	24 (0.012)	0.007	0.017
Rough	23 (0.012)	0.007	0.016
Less intense/weak	21 (0.011)	0.006	0.015
With faults	05 (0.003)	0.000	0.005
Stressed	03 (0.002)	0.000	0.003
With an effort	03 (0.002)	0.000	0.003
• **Vocal symptoms mode and time of onset**: sudden vocal symptoms were seen in 12 children, and gradual onset happened to 317 of them. Time of symptom onset was variable, from the beginning of the speech (n-72), less than one month (n-12), between one month and one year (n-171) or longer than one year (n-74).
• **Factors associated with a worsening in vocal quality, according to reports from the parents**: Table 3 lists the factors responsible for worsening in the vocal qualities as per reported by the parents, stressing excessive environmental noise, vocal abuse and having a flu.

**Table 3:** Confidence thresholds on the rate of response according to the voice-worsening-associated factor.

		Confidence threshold (95%)	
Associated Factors	Absolute frequency	Lower limit	Upper limit
Noisy environment	524 (0.262)	0.242	0.281
Excessive talking/Vocal abuse	313 (0.157)	0.141	0.172
Flu or colds	292 (0.146)	0.131	0.161
Exposure to cigarette smoking	226 (0.113)	0.099	0.127
Singing	110 (0.055)	0.045	0.065
Emotional problem	87 (0.043)	0.035	0.052
Sports with vocal abuse	41 (0.020)	0.014	0.026
Surgery with intubation	13 (0.007)	0.003	0.010
Parties	0(0.00)	0.00	0.00
• **Associated symptoms presented by the children**: among the symptoms associated to the vocal complaints, we stress: pulmonary allergic reactions (asthma, bronchitis) and nasal symptoms (sneezes, clear discharge, nasal pruritus), nasal obstruction of other causes (tonsil hypertrophy, keeping the nose blocked and complementary oral breathing), followed by auditory symptoms (Table 4).

**Table 4:** Confidence threshold of the associated symptoms presented by the children.

Associated symptoms	Absolute frequency (ratio)	Lower limit	Upper limit
Sneezing	318 (0.159)	0.142	0.175
Asthma/Bronchitis	300 (0.150)	0.134	0.165
Allergies	291 (0.145)	0.130	0.161
Headache	254 (0.127)	0.112	0.141
Blocked nose	203 (0.101)	0.088	0.114
Earache	166 (0.083)	0.070	0.095
Hearing difficulty	76 (0.0038)	0.029	0.046
Heartburn	53 (0.026)	0.019	0.033
Ear oozing	39 (0.019)	0.013	0.025
Sputum in the nose	32 (0.015)	0.010	0.021
Stomach ache	29 (0.014)	0.009	0.019
Tinnitus	29 (0.014)	0.009	0.019
Enlarged tonsils/ adenoids	14 (0.007)	0.003	0.011
Difficulty swallowing	3 (0.001)	0.000	0.003
• **Treatments administered, according to reports from the parents**: when questioned about the treatments administered to solve the vocal symptoms of their children, 232 (70.5%) of the parents answered they used medication only. Speech therapy to treat the vocal disorder was reported by 84 (25.5%) parents.
• **Results from the GRBASI scale assessments based on the G parameter scores**: the auditory-perceptual assessments showed that 707 (35.3%) children had the score 0 in the scale, pointing to the lack of vocal changes. Mild vocal deviations, scored 1 - seen in 1,065 children (53.3%) and moderate (score 2) in 228 (11.4%) children (Table 5).

**Table 5:** Voice change grade based on the G parameter score from the GRBASI scale, according to the age range (in years) and gender.

G score		0 – Absent		1 – Mild		2 – Moderate		Total General
Gender	Age range	N	%	N	%	N	%	
	4 - 6	102	44.8 a(1)A(2) β(3)	95	41,7 a A β	31	13.5 ab A α	228
Male	7 - 9	201	33.9 a A β	316	53,3 a A γ	76	12.8 a A γ	593
	10 - 12	68	36.6 a A β	101	54,3 a A γ	17	9.1 a A α	186
Total Male		371	18.5	512	25,6	124	6.2	1007
	4 - 6	91	38.4 a A β	127	53,6 a A γ	19	9.5 a A α	237
Female	7 - 9	185	33.2 a A β	313	56,1 a A γ	60	10.7 a A α	558
	10 - 12	60	30.3 a A β	113	57,1 a A γ	25	12.6 a A α	198
Total Female		336	16.8	553	27,65	104	5.2	993
Overall Total		707	35.3	1.065	53,3	228	11.4	2.000

(1) Two percentage frequencies followed by the same lower case letter do not differ as to the respective age ranges (lines), fixing the gender (*p*>0,05).

(2) Two percentage frequencies followed by the same upper case letter do not differ as to the respective genders (columns), fixing the age range (*p*>0,05).

(3) Two percentage frequencies followed by the same Greek letter do not differ as to the respective degrees of vocal change, fixing gender and age range (p>0,05).
• **Correlation between the results from the acoustic vocal and the auditory-perceptual analyses**: Table 6 depicts the mean fundamental frequency values and their correlation with the G score from the GRBASI scale. The f_0_ values reduced with the increase in age, in both genders. Among females, the children with a G score of 2 had the lowest f_0_ values. The other acoustic parameters (*jitter percentage, PPQ, APQ, shimmer percentage, NHR and SPI)* were higher in children with a G score of 2 (Table 6, Table 7, Table 8, Table 9, Table 10, Table 11, Table 12), confirming the high degree of agreement between both assessment techniques.

**Table 6:** Mean values and standard deviation (SD) of the fundamental frequency (f0) and correlation with the G parameter from the GRBASI scale, gender and the age range (in years).

G score		0-Absent	1-Mild	2-Moderate	Overall total
Gender	Age range	Mean± SD f_0_	Mean± SD f_0_	Mean ±SD f_0_	Mean±SD f_0_
	4 - 6	263.15 ± 33.69	254.97 ± 30.91	255.22 ± 29.17	258.71 ± 33.66 a^(1)^A^(2)^
Males	7 - 9	245.90 ± 28.09	245.01 ± 29.99	244.66 ± 32.56	245.24 ± 29.82 bA
	10 - 12	234.29 ± 19.81	228.06 ± 28.28	242.33 ± 29.17	231.64 ± 25.85 cA
Total Males		248.58 ± 30.16 Aα^(3^)	243.26531.00 Aα	246.83 ± 33.90 Aα	245.78 ± 31.25
	4 - 6	261.28 ± 33.02	255.76 ± 33.49	228.25 ± 33.46	256.43 ± 33.63 aA
Females	7 - 9	249.81 ± 31.57	243.88 ± 29.58	241.15 ± 26.72	245.45 ± 33.63 bA
	10 - 12	242.60 ± 20.35	242.96 ± 32.43	228.25 ± 12.40	240.71 ± 27.67 bB
Total Females		251.73 ± 30.99 Aα	246.42 ± 31.49 Aα	238.37 ± 26.13 Aß	247.12 ± 30.93
Overall Total		250.06 ± 30.57	244.94 ± 31.289	243.09 ± 30.95	246.45 ± 31.09

**Table 7:** Mean values and standard deviation (SD) of the Jitter % and correlation with the G parameter score from the GRBASI scale, gender and age range (in years).

G Score		0-Absent	1-Mild	2-Moderate
Gender	Age range	Mean ± SD Jitter	Mean ±SD Jitter %	Mean ± SD Jitter %
	4 to 6	0.729 ± 0.380 a^(1)^α^(2)^	1.455 ± 0.781 abß	2.988 ± 1.161 aγ
Males	7 to 9	0.827 ± 0.672 aα	1.464 ± 0.857 aß	2.282 ± 1.178 bγ
	10 to 12	0.842 ± 0.540 aα	1.685 ± 0.866 bß	4.251 ± 3.838 abγ
Total Males		0.802 ± 0.580	1.685 ± 0.849	2.650 ± 1.754
	4 to 6	0.765 ± 0.565 aα	1.742 ± 1.106 abß	3.484 ± 1.594 aγ
Females	7 to 9	0.765 ± 0.593 aα	1.549 ± 0.851 aß	2.593 ± 0.875 bγ
	10 to 12	0.814 ± 0.634 aα	1.975 ± 1.101 bß	2.734 ± 0.744 c abγ
Total Females		0.773 ± 0.593	1.685 ± 0.983	2.792 ± 1.069
Grand Total		0.788 ± 0.585	1.602 ± 0.926	2.713 ± 1.490

**Table 8:** Mean and standard deviation (SD) values of the PPQ (%) and correlation with the G parameter from the GRBASI scale, gender and the age range (in years).

G Score		0-Absent	1-Mild	2-Moderate
Gender	Age Range	Mean ± SD PPQ (%)	Mean ± SD PPQ (%)	Mean ± SD PPQ (%)
	4 - 6	0.422 ± 0.224 a^1^α^2^	0.896 ± 0.472 abß	1.959 ± 0.664 aγ
Males	7 - 9	0.476 ± 0.356 aα	0.849 ± 0.475 aß	1.501 ± 0.675 bγ
	10 - 12	0.496 ± 0.313 aα	1.005 ± 0.509 bß	2.052 ± 0.965 aγ
Total Males		0.465 ± 0.317	0.888 ± 0.484	1.691 ± 0.751
	4 - 6	0.457 ± 0.339 aα	1.004 ± 0.643 abß	2.068 ± 1.091 aγ
Females	7 - 9	0.445 ± 0.291 aα	0.944 ± 0.591 aß	1.688 ± 0.457 bγ
	10 - 12	0.471 ± 0.355 aα	1.081 ± 0.521 bß	1.785 ± 0.411 aγ
Total Females		0.453 ± 0.316	0.986 ± 0.592	1.781 ± 0.622
Grand Total		0.459 ± 0.318	0.939 ± 0.545	1.732 ± 0.695

**Table 9:** Mean and standard deviation (SD) values of the shimmer % and correlation with the G parameter score from the GRBASI scale, gender and age range (in years).

G Score		0-Absent	1-Mild	2-Moderate
Gender	Age Range	Mean ± SD % *Shimmer*	Mean ± SD % *Shimmer*	Mean ± SD % *Shimmer*
	4 - 6	3.544 ± 1.086 a^(1)^α^(3)^	4.864 ± 1.830 aß	7.929 ± 2.368 aγ
Males	7 - 9	3.711 ± 1.206 aα	4.526 ± 1.454 aß	7.435 ± 1.797 abγ
	10 - 12	3.947 ± 1.174 aα	4.681 ± 1.308 aß	6.176 ± 2.190 bγ
Total Males		3.708 ± 1.173 αA^(2)^	4.619 ± 1.507 ßA	7.386 ± 2.059 γA
	4 - 6	3.360 ± 1.124 aα	5.191 ± 2.201 aß	7.187 ± 2.999 aγ
Females	7 - 9	3.219 ± 1.056 aα	4.860 ± 1.742 aß	6.333 ± 2.347 abγ
	10 - 12	3.411 ± 0.969 aα	4.631 ± 1.752 aß	6.410 ± 2.895 bγ
Total Females		3.291 ± 1.060 αA	4.889 ± 1.865 ßB	6.507 ± 2.605 γA
Grand Total		3.510 ± 1.139	4.759 ± 1.707	6.985 ± 2.360

**Table 10:** Mean values and standard deviation (SD) of the APQ (in %) and correlation with the G parameter score from the GRBASI scale, gender and the age range (in years).

G Score		0-Absent	1-Mild	2-Moderate
Gender	Age Range	Mean ± SD APQ (%)	Mean ± SD APQ (%)	Mean ± SD APQ (%)
	4 - 6	2.531 ± 0.728 a^(1)^ α^(3)^	3.451 ± 1.180 aß	5.715 ± 1.805 aγ
Males	7 - 9	2.656 ± 0.813 aα	3.163 ± 1.005 bß	5.043 ± 1.168 bγ
	10 - 12	2.779 ± 0.836 aα	3.208 ± 0.821 bß	4.266 ± 1.481 bγ
Total Males		2.644 ± 0.797 A^(2)^α	3.226 ± 1.010 Aß	5.104 ± 1.451 Aγ
	4 - 6	2.390 ± 0.770 aα	3.630 ± 1.491 aß	5.010 ± 2.105 aγ
Females	7 - 9	2.354 ± 0.700 aα	3.389 ± 1.181 bß	4.411 ± 1.737 bγ
	10 - 12	2.434 ± 0.610 aα	3.224 ± 1.209 bß	4.434 ± 1.909 bγ
Total Females		2.378 ± 0.703 Bα	3.410 ± 1.269 Aß	4.526 ± 1.845 Aγ
Grand Total		2.518 ± 0.765	3.322 ± 1.156	4.841 ± 1.664

**Table 11:** Mean and standard deviation (SD) of the NHR and correlation with the G parameter score of the GRBASI scale, gender and age range (in years).

G Score		0-Absent	1-Mild	2-Moderate
Gender	Age Range	Mean ± SD NHR	Mean ± SD NHR	Mean ± SD NHR
	4 - 6	0.121 ± 0.012 a^(1)^α^(3)^	0.133 ± 0.034 aß	0.163 ± 0.057 aγ
Males	7 - 9	0.124 ± 0.017 aα	0.313 ± 1.361 aß	0.157 ± 0.053 bγ
	10 - 12	0.126 ± 0.015 aα	0.132 ± 0.025 aα	0.175 ± 0.067 abß
Total Males		0.124 ± 0.015 A^(2)^α	0.241 ± 1.060 Aß	0.274 ± 1.475 Aγ
	4 - 6	0.117 ± 0.016 aα	0.210 ± 0.666 aß	0.213 ± 0.112 aγ
Females	7 - 9	0.120 ± 0.017 aα	0.251 ± 1.447 aß	0.154 ± 0.046 bγ
	10 - 12	0.118 ± 0.016 aα	0.350 ± 1.183 aα	0.170 ± 0.061 abß
Total Females		0.119 ± 0.016 Aα	0.262 ± 1.251 Aß	0.169 ± 0.069 Aγ
Grand Total		0.122 ± 0.016	0.252 ± 1.165	0.227 ± 1.103

**Table 12:** Mean and Standard Deviation (SD) SPI values and correlation with the G parameter score from the GRBASI scale, gender and the age range (in years).

G Score		0-Absent	1-Mild	2-Moderate
Gender	Age Range	Mean ± SD	Mean ± SD	Mean ± SD
	4 - 6	4.375 ± 3.126 a^(1)^A^(2)^	4.894 ± 2.810 aA	5.739 ± 2.671 aA
Males	7 - 9	5.533 ± 3.758 aAB	6.801 ± 4.494 bB	6.661 ± 3.955 cA
	10 - 12	7.631 ± 5.530 aB	8.891 ± 5.357 bC	11.349 ± 4.642 cB
Total Males		5.597 ± 4.126	6.892 ± 4.608	6.943 ± 4.071
	4 - 6	4.076 ± 2.417 aA	5.721 ± 3.825 aA	6.031 ± 4.214 aA
Females	7 - 9	4.881 ± 3.737 aAB	5.792 ± 3.443 bB	7.379 ± 4.445 cA
	10 - 12	4.602 ± 1.995 aB	7.998 ± 4.224 bC	8.803 ± 5.894 cB
Total Females		4.614 ± 3.132	6.242 ± 3.812	7.449 ± 4.815
Grand Total		5.135 ± 3.723	6.547 ± 4.215	7.166 ± 4.414
• **Results from the videolaryngoscopy exams**: the endoscopic exams were done in 259 children, from which 73 did not have vocal symptoms or changes in auditory-perceptual vocal assessments. Endoscopic exams were normal in 115 children (44.4%); vocal nodules were the most often diagnosed laryngeal lesions (Table 13; Figs. 3 and 4). Direct laryngoscopy exam was indicated in 20 children; 13 of them were submitted to laryngeal microsurgery for the removal of nodules and six for cyst removal, all of them being epidermal. The only case of vocal sulcus diagnosed in children was also submitted to direct laryngoscopy exam in order to confirm the diagnosis; nonetheless no procedure has been carried out so far. The parents of all the children who were submitted to this exam signed the informed consent form, authorizing the procedure.

**Table 13:** Results from the video laryngoscopies.

G Score	0 – Absent		1 – Mild		2 – Moderate		Total	
G Score	Fem N	Male N	Fem N	Male N	Fem N	Male N	N	(%)
Laryngeal diagnosis								
Normal test	21	19	37	24	9	5	115	44,4
Vocal nodules	2	2	28	31	8	10	81	31,3
Mucosal thickening	7	11	2	6	2	4	32	12,3
Edema and/or congestion	5	4	3	2	0	1	15	5,8
Vocal cysts	1	1	1	3	1	2	9	3,5
Undetermined Diagnosis	0	0	1	1	1	2	5	1,9
Sulcus	0	0	1	0	0	0	1	0,4
Bridge	0	0	0	1	0	0	1	0,4
Total	36	37	73	68	21	24	259	100,0

**Figure 3:**
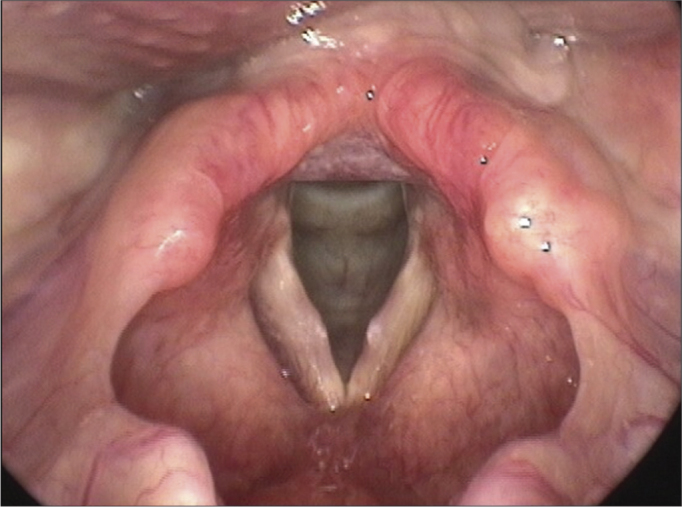
Bilateral vocal nodules.

**Figure 4:**
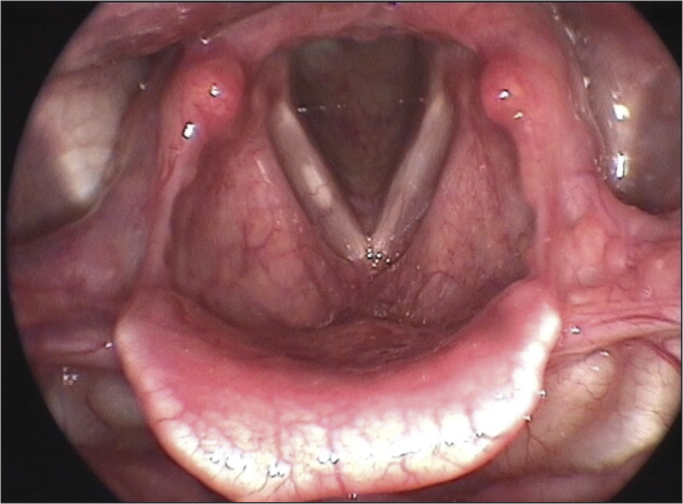
Right vocal fold cyst.

## DISCUSSION

The 2,000 children in this study were further broken down by age range, because of the constant growth and development of the infantile larynx, responsible for gradual changes in vocal quality, which can be more pronounced at 12 years of age. The most evident vocal changes happen to boys and are due to high levels of systemic hormones during adolescence^16^,^17^. Therefore, we chose to exclude two older children because of the proximity to this period of hormonal-caused changes. There was a better compliance of children in the age range between 7 and 9 years from the selected school.

Numerous methodologies are used in epidemiological studies about voice disorders. One of them is to calculate the prevalence of dysphonia based solely on the reports from the parents. In these cases, it is recommended that the questionnaires be created in layman's terms, with clear and objective questions. Parents must also be informed about the concepts of normal and changed voice, thus they must be instructed on how to fill out the questionnaires, and we did exactly this in the present study. It is not advisable to factor sporadic vocal symptoms in the dysphonia prevalence calculations, because of how frequent they are found in the pediatric population during sports or leisure activities, or even during upper airway infection spells. On the other hand, permanent symptoms indicate a persistence of altered vocal patterns – of varied causes, both functional and organic. The functional ones stem from the phonatory overload and are characterized by an increase in voice intensity, effort and stress during utterance, and such phonatory pattern favors the development of laryngeal lesions.

Dysphonia prevalence indices reported in the literature vary between 4.4% and 30.3%^3^, ^4^, ^5^, ^6^, ^7^, ^8^- values which are close to the ones hereby found, between 6% and 7%, calculated based on the report of the parents, were presented by Leeper et al.^5^ in their analysis of the vocal quality of 1,481 children; and atypical voices were found in 104 of them (7%). Pastrelo & Behlau^8^ analyzed the voices of 363 children, and they found dysphonia values mildly lower than these ones, around 4.4%. Duff et al.^6^ investigated the prevalence of vocal disorders in 2,445 children (girls-1,199; boys -1,246) between 2 and 6 years of age, and they found 95 (3.9%) children with atypical voices. Higher percentage values were reported by Yari et al.^4^ – around 13.8%, in a vocal assessment of 1,549 children. These discrepancies among studies may be minimized with the inclusion of other assessment methods in the studies. Carding et al.^10^ interviewed the parents of 7,389 8-year-old children and found that 11.6% of them had vocal problems; compared to 6% of atypical voices found by speech therapists, reinforcing the importance of using different assessment methods.

In our study, most of the parents reported vocal symptoms, such as hoarseness and fatigue after vocal abuse. Vocal abuse, especially in a noisy environment, was indicated as the most important worsening factor associated with vocal symptoms. Vocal abuse, besides bearing a high phonatory demand, is, in many cases, followed by an increase in voice intensity, especially in children, and hyperfunctional peaks with muscle-skeletal stress. This phonatory pattern causes the traumatic collision of the vocal folds and then the development of laryngeal lesions, such as vocal nodules^2^,^3^. In a study involving 137 children with dysphonia, Connelly et al.^18^ found vocal abuse in 62 of them (45%). Pediatric dysphonia is also worsen by respiratory allergies and nasal obstruction, which were also stressed in the present study. Some authors reported an important improvement in acoustic vocal values in patients submitted to tonsil surgery, when compared to preoperative analyses^12^,^13^.

The drug treatment for the vocal symptoms most parents reported is indicated in acute upper airway infections. However, vocal training is an integral part of chronic vocal disorder treatment, for they stem from inadequate voice use^3^. Speech therapy was followed by a smaller number of children, and it may be associated with the difficulties to get to specialized treatment, low family income and a lack of diagnosis.

Auditory-perceptual analyses using the GRBASI scale found mild and moderate changes in 53.3% and 11.4% of the children (*p*<0,05), respectively. Mild changes in the B (Breathiness), S (Stress) and I (Instability) parameters are commonly seen in children's voices and must not be considered pathological. Breathiness (B) values may arise from the glottic configuration of the pediatric larynx, with posterior triangular slit^19^. Stress (S) may point to a hyperfunctional status and be identified in the voices of excited children telling a story. Based on the aforementioned remarks, the present study appreciated the vocal changes scored only on the G parameter above 1 for the calculation of the dysphonia prevalence; which represented 11.4% of the cases; and such percentage was 1.8 times higher than the one reported in the parents reports – around 6.15%. Therefore, we stress the importance of using more than one method of assessment.

Acoustic analyses have shown that the f_0_ values reduced as age increased; and the lowest values were seen in the children with a G score of 2. Such f_0_ behavior was previously reported by many authors who study children without vocal symptoms, determining normal acoustic values in the different age ranges, going from 257Hz and 275Hz at 4 to 5 years; to 234Hz and 222 Hz at 10 and 11 years of age, among girls and boys, respectively^19^. F_0_ is considered an important acoustic parameter, maintaining a direct relationship with length, stress, stiffness, vocal fold mass and subglottic pressure^20^.

The other acoustic parameters (% *jitter* PPQ, % *shimmer*, APQ, NHR and SPI) are higher in children with a G score of 2. *Jitter* and PPQ score the number of aperiodic cycles, representing important indicators of the degree of vocal involvement^3^. *Shimmer* and APQ express the irregularities of the sound wave amplitude, and correlates to noise upon utterance (hoarseness) and breathiness. These parameters may be changed in laryngeal lesions, with an increase in vocal fold mass because of vibratory irregularities^21^,^22^. NHR associates the harmonic component with the noise in the sound wave, and the SPI indicates the mild phonation coefficient, being high in the excess of breathiness upon utterance, as it happens to glottic slits and hyperfunctional dysphonias^3^,^22^.

The videolaryngoscopy exams were done in 259 children, of whom 73 were asymptomatic and received a score of 0 in the perceptual assessment. The 186 remaining children had vocal symptoms or changes of different intensity seen upon the GRBASI scale. If we consider a total of 329 children with vocal symptoms, the compliance rate to endoscopic exams was 56.5%; a marked percentage; however, far from ideal. This rate is justified by the need of the parents to commute to the hospital in order to undergo the exam and, consequently, skip work, besides the lack of parental knowledge as to the technique of the exam and the little collaboration of the children.

Videolaryngoscopy exams were normal in many children with vocal symptoms; thus, characterizing the functional dysphonia cases, responsible for most of the infantile dysphonia. They stem from the inadequate and exaggerated use of voice, in the absence of structural or organic lesion on the vocal folds^1^. Auditory-perceptual assessment is valuable in such cases, as well as the nasofibroscopy exam, which allows for the vocal dynamic analysis in a natural and spontaneous fashion. Hyperfunctional dysphonia is characterized by an exaggerated contraction of the intrinsic and extrinsic laryngeal muscles, resulting in a traumatic collision of the vocal folds and a posterior triangular glottic slit. Videolaryngoscopy may not show structural lesions on the vocal folds; nonetheless, laryngeal edema and mucosal congestion are common findings. The development of lesions on the laryngeal mucosa, such as vocal nodules (Fig.3), are also frequent, especially among boys between 5 and 10 years of age, and there is a gender inversion after puberty, which was highlighted in the endoscopic exams of the present study^2^,^3^,^8^. These lesions were diagnosed in 81 children of the present study, and there is a predominance in boys.

Vocal cysts, sulci and mucosal bridges were also diagnosed in the present study - Pontes et al.^23^ called them minimum laryngeal structural lesions. They correspond to mild histological derangements of the vocal fold epithelial cover, able to impair the vibratory cycle. These lesions may course with vocal symptoms since the first years of life – as per reported by some parents in the questionnaire; and they have been diagnosed increasingly earlier in children, thanks to the development in videolaryngo-stroboscopy devices. Cysts are the most frequent lesions, and they may be classified as epidermoid or mucosal; being considered the main causes of hoarseness in children, after nodules and thickenings^24^.

The joint analyses of the results from the present study indicated an agreement between the two methods utilized in the vocal analysis (acoustic and auditory-perceptual). Nonetheless, there was a disagreement between the information in the parents' questionnaires and the auditory-perceptual speech assessments, pointing to the importance of associating many methodology assessment tools and the low credibility regarding the information provided by the family, even after a thorough instruction on how to fill out the questionnaire.

## CONCLUSIONS

Parental judgment indicated a dysphonia prevalence rate of 6.15% and the vocal auditory-perceptual analyses pointed to 11.4%; therefore showing a disagreement between both methods of assessment. On the other hand, the acoustic measures kept a direct relationship with the auditory-perceptual analyses scores. The vocal symptoms reported by the parents were associated with phonatory overload, and the predisposing factors were: excessive environmental noise, allergy and nasal obstruction. The most frequently diagnosed laryngeal lesions in the videolaryngoscopic exam were: vocal nodules, mucosal thickening and inflammatory processes.

## ACKNOWLEDGEMENTS

We thank FAPESP - Research Support Agency, Capes and the Brazilian Association of Otorhinolaryngology and Neck and Facial Surgery for their financial support.
